# Loss of Myeloid-Specific TGF-β Signaling Decreases CTHRC1 to Downregulate bFGF and the Development of H1993-Induced Osteolytic Bone Lesions

**DOI:** 10.3390/cancers10120463

**Published:** 2018-11-22

**Authors:** Sourik S. Ganguly, Paul G. Daft, Jingchen Cao, Xiangqi Meng, Zhendong A. Zhong, Alexandra Vander Ark, Austin Meadows, Zach Madaj, Bart Williams, Xiaohong Li

**Affiliations:** 1 Program for Skeletal Disease and Tumor Microenvironment, Center for Cancer and Cell Biology, Grand Rapids, MI 49503, USA; Sourik.Ganguly@vai.org (S.S.G.); paul.daft@gmail.com (P.G.D.); jingchen.cao@vai.org (J.C.); jzmxq@hotmail.com (X.M.); alex.zhong@vai.org (Z.A.Z.); Alexandra.VanderArk@vai.org (A.V.A.); meadowau@mail.gvsu.edu (A.M.); bart.williams@vai.org (B.W.); 2 Bioinformatics & biostatistics Core, Van Andel Research Institute, Grand Rapids, MI 49503, USA; Zachary.madaj@vai.org

**Keywords:** TGF-β signaling, TGFBR2, bFGF, CTHRC1, Wnt/β-catenin, NSCLC bone metastasis, myeloid lineage cells, osteoclasts, osteoblasts

## Abstract

The role of myeloid cell-specific TGF-β signaling in non-small-cell lung cancer (NSCLC)-induced osteolytic bone lesion development is unknown. We used a genetically engineered mouse model, *Tgfbr2^LysMCre^* knockout (KO), which has a loss of TGF-β signaling specifically in myeloid lineage cells, and we found that the area of H1993 cell-induced osteolytic bone lesions was decreased in *Tgfbr2^LysMCre^* KO mice, relative to the area in control littermates. The bone lesion areas were correlated with tumor cell proliferation, angiogenesis, and osteoclastogenesis in the microenvironment. The smaller bone lesion area was partially rescued by bFGF, which was expressed by osteoblasts. Interestingly, bFGF was able to rescue the osteoclastogenesis, but not the tumor cell proliferation or angiogenesis. We then focused on identifying osteoclast factors that regulate bFGF expression in osteoblasts. We found that the expression and secretion of CTHRC1 was downregulated in osteoclasts from *Tgfbr2^LysMCre^* KO mice; CTHRC1 was able to promote bFGF expression in osteoblasts, possibly through the Wnt/β-catenin pathway. Functionally, bFGF stimulated osteoclastogenesis and inhibited osteoblastogenesis, but had no effect on H1993 cell proliferation. On the other hand, CTHRC1 promoted osteoblastogenesis and H1993 cell proliferation. Together, our data show that myeloid-specific TGF-β signaling promoted osteolytic bone lesion development and bFGF expression in osteoblasts; that osteoclast-secreted CTHRC1 stimulated bFGF expression in osteoblasts in a paracrine manner; and that CTHRC1 and bFGF had different cell-specific functions that contributed to bone lesion development.

## 1. Introduction

Advanced prostate, breast, and lung cancers often metastasize to bone. Bone metastases produce skeletal-related events, including bone pain, fracture, spinal cord compression, and hypercalcemia [1,2]. The metastasized cancer cells dysregulate osteoclasts (bone resorption) and osteoblasts (bone formation), resulting in the development of bone lesions. Some 20%–40% of patients with non-small-cell lung cancer (NSCLC) develop bone metastases, and these patients have a median survival rate of one year [3,4,5,6]. Metastatic NSCLC causes mainly osteolytic bone lesions and excessive bone resorption by osteoclasts [7].

The bone microenvironment is rich with growth factors and cytokines, including transforming growth factor-β (TGF-β) and the fibroblast growth factors (FGFs), which promote the proliferation of metastasized cancer cells, as well as the proliferation and differentiation of osteoclasts and osteoblasts [8,9,10,11]. Canonical TGF-β signaling occurs when ligand binds to the TGF-β type II receptor (TGFBR2), which then recruits and phosphorylates TGFBR1. In turn, phosphorylated TGFBR1 phosphorylates downstream SMAD2 and SMAD3, which recruit SMAD4. This SMAD complex translocates to the nucleus and stimulates the transcription of TGF-β target genes [12]. The effects of TGF-β signaling, either suppressing or promoting tumor growth, are highly context-dependent spatially and temporally [13,14].

In NSCLC, TGF-β and its signaling pathways have been correlated with poor prognosis, and have been implicated in promoting cancer progression [15]. In the tumor microenvironment, myeloid-specific TGF-β signaling is necessary for soft organ metastasis in experimental mouse models [16]. Myeloid cells are crucial players in tumor progression, inhibiting host immune surveillance which favors cancer metastasis [17,18,19]. Our previous studies showed that TGF-β signaling in osteoclasts, which are myeloid lineage cells, promotes osteolytic bone lesions in prostate or breast cancer [20,21]. However, the source(s) of differences in osteolytic bone lesion development in NSCLC are not known.

CTHRC1 (collagen triple helix repeat-containing 1), a 30 kDa glycosylated protein, was first identified in the healing process of injured arteries [22]. There has been a growing enthusiasm for targeting CTHRC1 in the progression and metastasis of various cancers, including NSCLC [23,24,25,26]. For example, CTHRC1 expression at both mRNA and protein levels was significantly higher in NSCLC tumor cells than in adjacent noncancerous tissues [27]; increased serum CTHRC1 has been found in NSCLC patients and positively correlated with metastasis [28]; and CTHRC1 promotes NSCLC cell proliferation and motility, and could be a biomarker of poor prognosis for NSCLC patients [27]. On the other hand, CTHRC1 is expressed in a variety of cell types, including osteoclasts [23], and CTHRC1 promotes either the Wnt/PCP or the Wnt/β-catenin pathways in a context-dependent manner [29,30,31]. However, the paracrine effect of osteoclast-secreted CTHRC1 in cancer-induced bone lesion development is unknown. In this study, we demonstrated that the development of osteolytic bone lesions after injection of H1993 cells was inhibited in mice by deletion of the *Tgfbr2* gene in myeloid lineage cells (*Tgfbr2^LysMCre^* knockout (KO)), and this inhibition could be partially rescued by bFGF. We also show that CTHRC1 stimulated the expression of bFGF in osteoblasts through increasing Wnt/β-catenin signaling, and the cell-specific roles of bFGF and CTHRC1 in cancer and bone cells.

## 2. Results

### 2.1. Osteolytic Bone Lesions Were Inhibited in Tgfbr2^LysMCre^ KO Mice

**Figure 1:**
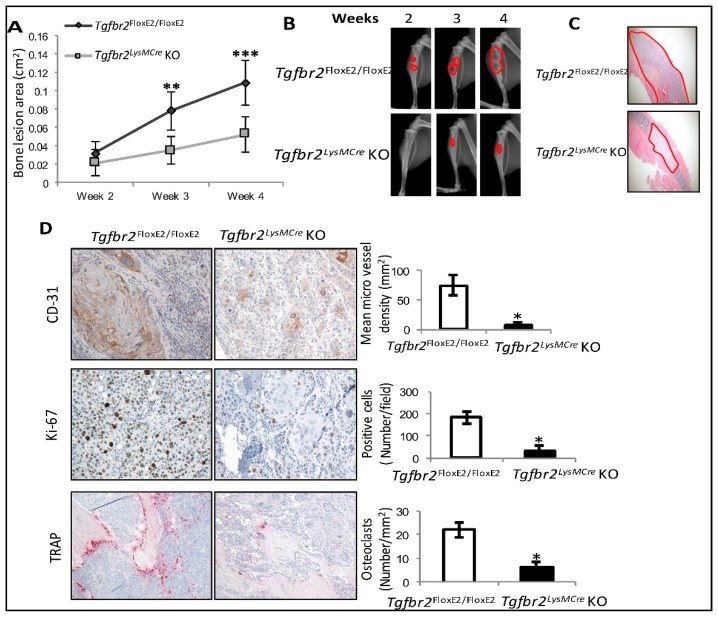
H1993-induced bone lesions were inhibited in *Tgfbr2^LysMCre^* knockout (KO) mice. (**A**) Quantification of bone lesion areas in the control and KO mouse tibiae. (**B**) Representative bone lesions (red outlines) shown by weekly X-ray images. (**C**) Hematoxylin and eosin (H&E) staining images (4×) confirm tumor growth in the mouse tibiae (red outlines) at 4 weeks (wk) post-injection. (**D**) Representative mouse tibiae at 4 wk post-injection, stained for CD31 (20×), Ki67 (20×), or TRAP (10×). Mean ± SD, *n* ≥ 10 per group. *** *p* < 0.001, ** *p* < 0.01, * *p* ≤ 0.05, by linear mixed-effect models for bone lesions and Student’s *t*-test for paired comparison.

In the absence of cancer cells, there are no differences between tibiae from *Tgfbr2^floxE2/floxE2^* and *Tgfbr2^LysMCre^* KO mice, as analyzed by μCT and bone histomorphometry, nor are there differences in osteoclast differentiation from bone marrow [20]. In this study, H1993 NSCLC cells were injected into the tibiae of *Tgfbr2^floxE2/floxE2^* and *Tgfbr2^LysMCre^* KO mice, and osteolytic bone lesions subsequently developed. We found that the osteolytic lesion development was inhibited in the *Tgfbr2^LysMCre^* KO mice, with significantly smaller lesion areas at 3 and 4 weeks after injection (Figure 1A,B). The growth of cancer cells in the lesion areas was confirmed using histology staining (Figure 1C).

To investigate the bone lesions at the cellular and molecular levels, we performed IHC staining in H1993-injected tibiae for Ki67 as a cell proliferation marker, CD31 as an endothelial cell marker, and tartrate-resistant acid phosphatase (TRAP) staining as an osteoclast marker. We found decreases of Ki67-, CD31-, and TRAP-positive cells in the H1993-injected tibiae from *Tgfbr2^LysMCre^* KO mice, relative to those from *Tgfbr2^floxE2/floxE2^* mice (Figure 1D). These results indicated that myeloid-specific TGF-β signaling contributed to the H1993-induced osteolytic bone lesion development, and the bone lesions were correlated with increases of cancer cell proliferation, angiogenesis, and osteoclastogenesis in the bone microenvironment.

### 2.2. Myeloid-Specific TGF-β Signaling Promoted H1993-Induced Bone Lesions and bFGF Expression

Our previous study showed that increased bFGF partially mediates the myeloid-specific, TGF-β signaling-induced, osteolytic bone lesions in breast cancer [20,21]. In this study, increased bFGF protein were found in H1993-injected (but not mock-injected) tibiae from *Tgfbr2^LysMCre^* KO, relative to bFGF in control mice (Figure S1A). Using species-specific qRT-PCR, we further showed that it was the mouse-specific, but not the human-specific, bFGF that was decreased at the transcript level in H1993-injected tibiae from *Tgfbr2^LysMCre^* KO mice, relative to controls (Figure S1B).

**Figure 2:**
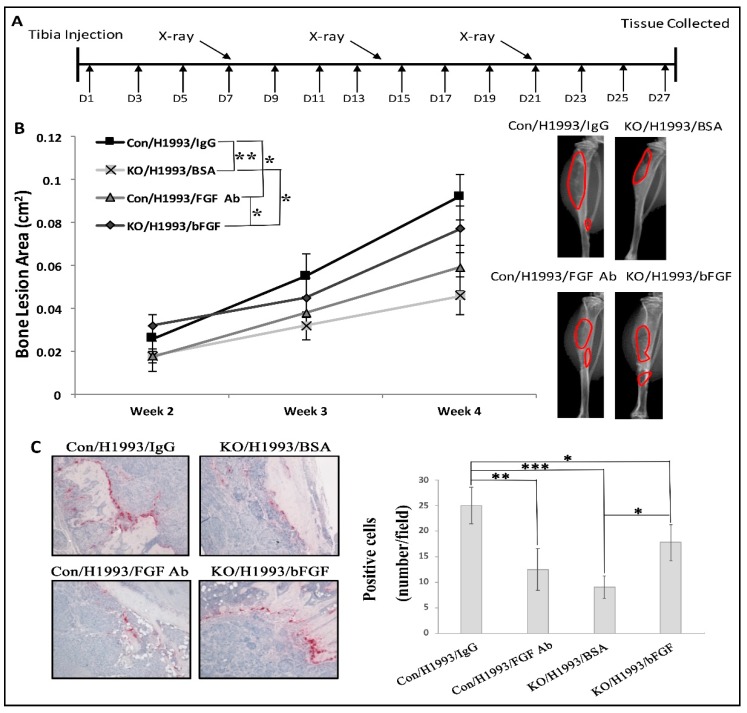
bFGF rescued H1993-induced lytic lesion development in *Tgfbr2^LysMCre^* KO mice. (**A**) The schedule of drug treatment, X-ray image acquisition, and end-point tissue collection. (**B**) Quantification of bone lesion areas and representative X-ray images at 4 wk post-injection. (**C**) Representative TRAP staining (20×). Mean ± SD, *n* ≥ 7 per group. *** *p* < 0.001, ** *p* < 0.01, * *p* ≤ 0.05, by linear mixed-effect models for bone lesions or by ANOVA for quantification of TRAP.

To test the functional role of bFGF, we performed rescue experiments (Figure 2A). We found consistently smaller bone lesions in *Tgfbr2^LysMCre^* KO tibiae relative to those in *Tgfbr2^floxE2/floxE2^* tibiae. Treatment with neutralizing bFGF antibody (bFGF Ab) significantly decreased H1993-induced bone lesion area in *Tgfbr2^floxE2/floxE2^* mice, and treatment with recombinant bFGF significantly increased lesion area in *Tgfbr2^LysMCre^* KO mice (Figure 2B). bFGF signals through the FGF receptors and activates the downstream MAPK (mitogen-activated protein kinase), PI3K (phosphoinositide 3-kinase), or PLCγ (phospholipase C gamma) pathways which, in turn, could promote cell proliferation, survival, and motility, respectively [32]. Western blot experiments showed that H1993-injected tibiae from *Tgfbr2^LysMCre^* KO had lower levels of pERK, but had no difference in p-AKT expression, relative to *Tgfbr2^floxE2/floxE2^* mice (Figure S1C). Note that the antibodies used were not species-specific, so the intensities of the bands reflected protein expression from both H1993 NSCLC cells and the mouse bone cells. These data suggested that, in H1993-induced bone lesions, bFGF likely signals through FGFR1 and activates the downstream MAPK/ERK pathway, but not the PI3K/AKT pathway.

Using IHC, we found that the bone lesion areas were positively correlated with the number of cancer-associated osteoclasts (Figure 2C). However, neither the proliferation of H1993 cells nor angiogenesis was affected by FGF Ab treatment in *Tgfbr2^floxE2/floxE2^* mice or by bFGF treatment in *Tgfbr2^LysMCre^* KO mice, as indicated by Ki67 or CD31 staining, respectively (Figure S2). These data suggest that myeloid-specific TGF-β signaling promoted H1993-induced bone lesion development, in part, in a bFGF-dependent manner; the bone lesion areas were positively correlated with osteoclastogenesis. We further performed in vitro osteoclast differentiation from mouse bone marrow, and bFGF did indeed increase osteoclastogenesis (Figure S3). TRAP staining showed the differentiated osteoclasts, and crystal violet staining showed cell proliferation. No differences between *Tgfbr2^floxE2/floxE2^* and *Tgfbr2^LysMCre^* KO mice were found, possibly due to the lack of TGF-β induction in the culture medium.

### 2.3. Loss of TGFBR2 in Osteoclasts Decreased the Expression and Secretion of CTHRC1 

**Figure 3:**
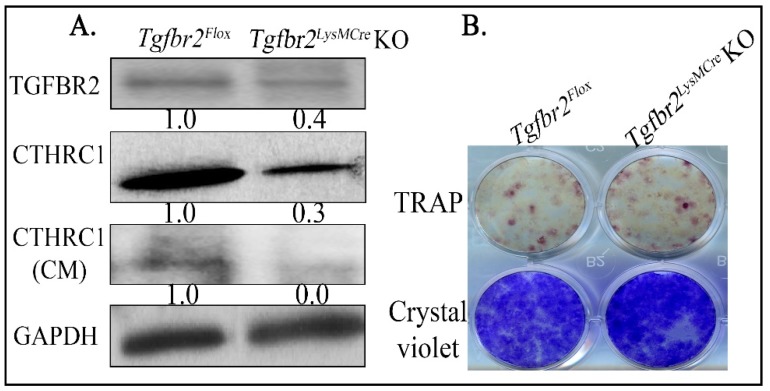
Osteoclasts from *Tgfbr2^LysMCre^* KO mice had lesser expression of CTHRC1 relative to littermate controls. (**A**) Primary osteoclasts were cultured from *Tgfbr2^LysMCre^* KOs and littermate controls. The total cell lysis or conditioned media (CM, after concentration) were subjected to Western blotting. (**B**) Representative images of differentiated osteoclasts from the bone marrow of *Tgfbr2^floxE2/floxE2^* or *Tgfbr2^LysMCre^* KO mice. Numbers under each blot are relative quantification of the densities normalized to GAPDH. Experiments were repeated at least three times with same results. More than 12 mice per group were used for collecting bone marrow.

bFGF is expressed in mesenchymal lineage cells, such as osteoblasts [20,21]. The regulation of bFGF expression in osteoblasts by myeloid-specific TGF-β signaling is likely via a secreted factor, such as clastokines [33], from osteoclasts. Therefore, we tested whether CTHRC1, a known clastokine [24,30] and a TGF-β target [34], regulates bFGF. Relative to *Tgfbr2^floxE2/floxE2^* mice, we found *Tgfbr2^LysMCre^* KO mice had significantly lower CTHRC1 protein expression and secretion in primary cultured osteoclasts (Figure 3A), which were differentiated from bone marrow collected from the *Tgfbr2^floxE2/floxE2^* and *Tgfbr2^LysMCre^* KO mice. Note that decreased, but not complete loss of, TGFBR2 expression was detected, as the differentiation of osteoclasts from mouse bone marrow cells was less than 50% (Figure 3B). Consistent with our previous report [21], no significant difference in osteoclast differentiation was found between floxed and KO mice (Figure 3B). We also tested the reported clastokines [33] at the transcript level. Among those, CTHRC1 and SOST were decreased in primary osteoclasts cultured from *Tgfbr2^LysMCre^* KOs and littermate controls (Figure S4). However, we were not able to detect and confirm the change of SOST at protein level using Western blot.

### 2.4. CTHRC1 Induced the Expression of bFGF in Osteoblasts and Osteoblastogenesis

**Figure 4:**
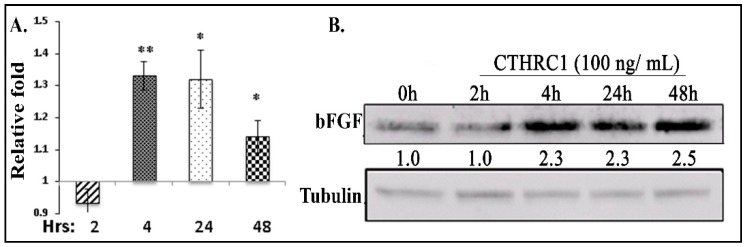
Effects of CTHRC1 in primary osteoblasts. Primary osteoblasts were treated with recombinant CTHRC1 or bFGF. CTHRC1 promoted bFGF expression at the mRNA level (**A**) and the protein level (**B**). The relative expression levels of mouse-specific bFGF at various time points were compared to the 0 h group using qRT-PCR (*n* = 3). Whole-cell lysates were used for Western blots. Numbers under each blot were relative quantification of the densities normalized to tubulin. Experiments were repeated at least three times with the same results. * *p* ≤ 0.05, ** *p* ≤ 0.01, by Student’s *t*-test. More than six mice were used for collecting bone marrow.

We treated primary osteoblasts with recombinant CTHRC1, and found that the expression of bFGF was increased at 4 h post-treatment at both the mRNA (Figure 4A) and protein levels (Figure 4B). On the other hand, CTHRC1 treatment did not stimulate the expression of bFGF protein in H1993 cells (Figure S5A,B), supporting the idea that murine bFGF, but not human bFGF, decreased in H1993-injected *Tgfbr2^LysMCre^* KO mouse tibiae (see Figure S1A,B). Consistent with CTHRC1 inducing NSCLC cell proliferation in our hand (Figure S5C) and a previous publication [27], H1993 cell viability, in vitro, was indeed decreased when the cells were incubated with conditioned medium of cultured osteoclasts; the medium of osteoclasts from *Tgfbr2^LysMCre^* KO mice had a lesser amount of CTHRC1 than that from *Tgfbr2^floxE2/floxE2^* mice (Figure S5D).

**Figure 5:**
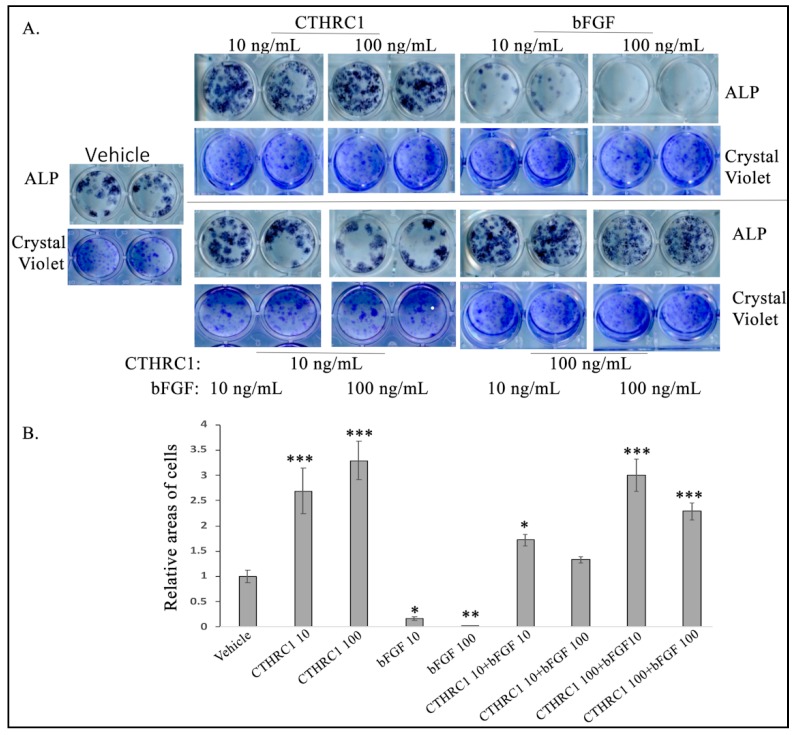
CTHRC1 stimulated, but bFGF inhibited, osteoblastogenesis. (**A**) Representative images of alkaline phosphatase (ALP) or crystal violet staining. (**B**) Relative quantification of the areas of ALP-positive cells normalized by their respective areas of total live cells as indicated by crystal violet staining. Experiments were repeated at least three times with the same results. * *p* ≤ 0.05, by Student’s *t*-test. More than 12 mice were used for collecting bone marrows. *** *p* < 0.001, ** *p* < 0.01, * *p* ≤ 0.05, by ANOVA. Other significant differences were found *p* < 0.001 in CTHRC1 10 vs. bFGF 10, CTHRC1 10 vs. bFGF 100, CTHRC1 10 vs. CTHRC1 10+bFGF 100, CTHRC1 100 vs. bFGF 10, CTHRC1 100 vs. bFGF 100, CTHRC1 100 vs. CTHRC1 10+bFGF 10, CTHRC1 100 vs. CTHRC1 10+bFGF 100; bFGF 10 vs. CTHRC1 100, bFGF 10 vs. CTHRC1 10+bFGF 10, bFGF 10 vs. CTHRC1 10+bFGF 100, bFGF 10 vs. CTHRC1 100+bFGF 10, bFGF 10 vs. CTHRC1 100 + bFGF100, bFGF 100 vs. CTHRC1 10 +bFGF 10, bFGF 100 vs. CTHRC1 10+bFGF 100, bFGF 100 vs. CTHRC1 100+bFGF 10, bFGF 100 vs. CTHRC1 100+bFGF 100, CTHRC1 10+bFGF 10 vs. CTHRC1 100+bFGF 100, CTHRC1 10+bFGF 100 vs. CTHRC1 100+bFGF 10; *p* < 0.01 in CTHRC1 10 vs. CTHRC1 10+bFGF 10, CTHRC1 100 vs. CTHRC1 100+bFGF 100, CTHRC1 10+bFGF 100 vs. CTHRC1 100+bFGF 100; *p* ≤ 0.05 in CTHRC1 100+bFGF 10 vs. CTHRC1 100 +bFGF 100.

We also tested the functional roles of CTHRC1 and bFGF in osteoblastogenesis. Consistently with previous publications [21,24], CTHRC1 alone induced, but bFGF alone inhibited, osteoblastogenesis (Figure 5). When the treatments were combined, the effects on osteoblastogenesis were dose-dependent. For example, 10 ng/mL CTHRC1 was able to rescue, and 100 ng/ml CTHRC1 was able to eliminate, the inhibition effects of bFGF (Figure 5). Altogether, these data suggest that CTRHC1 can induce bFGF expression in osteoblasts and osteoblastogenesis and that TGF-β signaling in osteoclasts might be required for the expression and secretion of CTHRC1 in osteoclasts. Furthermore, CTHRC1 and bFGF had opposite effects on osteoblastogenesis, but CTHRC1 could eliminate the effect of bFGF dose-dependently.

### 2.5. CTHRC1 Promoted bFGF Expression in a Wnt/β-Catenin-Dependent Manner

**Figure 6:**
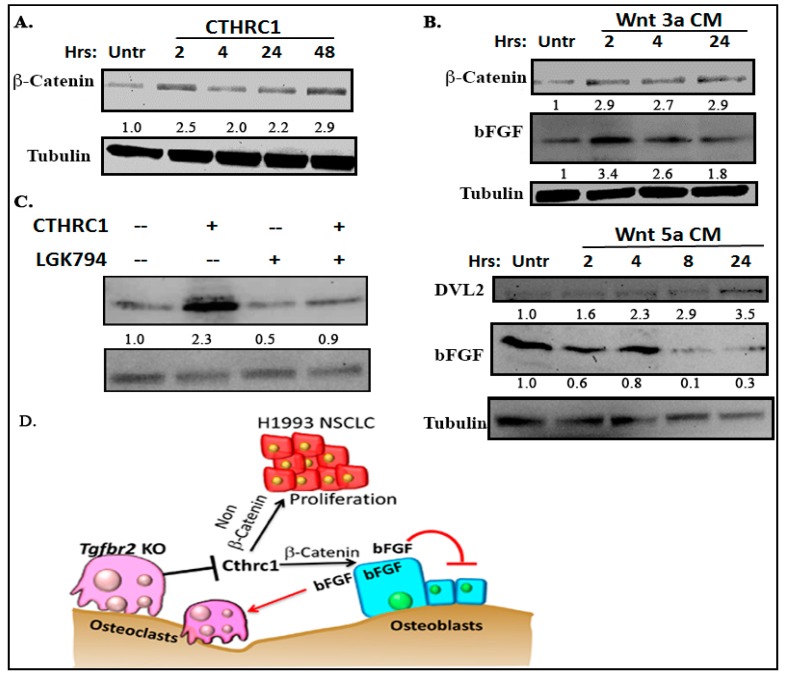
CTHRC1 promoted bFGF in a Wnt/β-catenin-dependent manner. Western blots of primary osteoblasts that were treated for various times with CTHRC1 (**A**), Wnt3a-conditioned medium (**B**, upper panel), Wnt5a-conditioned medium (**B**, lower panel), or CTHRC1 with LGK-974 (1 nM/mL) (**C**). The changes of β-catenin and DVL-2 indicate the activation of canonical Wnt pathway, such as by CTHRC1 or Wnt3a, and of the non-canonical Wnt pathway, such as by Wnt5a, respectively. Numbers under each blot are relative quantification of the densities normalized to tubulin. Total cell lysates were used for blotting. Experiments were repeated at least three times with same results. More than 12 mice were used for collecting bone marrows. (**D**) Working model. Loss of TGFBR2 in osteoclasts decreased the expression and secretion of CTHRC1, which stimulated bFGF, possibly through promoting Wnt/β-catenin signaling. Functionally, bFGF stimulated osteoclastogenesis, but inhibited osteoblastogenesis. On the other hand, CTHRC1 could stimulate H1993 cell proliferation, possibly through non-β-catenin signaling and osteoblastogenesis.

CTHRC1 promotes Wnt signaling through either β-catenin or non-β-catenin pathways [27,29,31,35]. We found that CTHRC1 promoted the expression of β-catenin (Figure 6A). Conditioned medium containing Wnt3a (an activator of the canonical Wnt pathway, acquired from a culture of L-Wnt-3a fibroblasts), but not medium containing Wnt5a (an activator of the noncanonical Wnt pathway), promoted the expression of bFGF in osteoblasts (Figure 6B). On the contrary, Wnt5a-conditioned medium decreased bFGF in osteoblasts at 8 h and 24 h after the medium was added. DVL-2 was used as an indicator of non-β-catenin signaling activation. To confirm this finding, we pretreated primary osteoblasts with LGK-974 24 h to block Wnt before treatment with CTHRC1. LGK-974 is a porcupine inhibitor that inhibits Wnt’s secretion [36]. The prior treatment with LGK-974 blocked CTRHC1-induced bFGF expression (Figure 6C), which suggests that CTHRC1 promoted bFGF expression in a Wnt/β-catenin-dependent manner (Figure 6D).

## 3. Discussion

Our study shows that TGF-β signaling in myeloid lineage cells promoted the development of H1993-induced osteolytic bone lesions. The increase bone lesion development was at least partially mediated by bFGF. These findings aligned with our previous studies on osteolytic bone lesion development in other cancers [20,21]. Interestingly, bFGF mediated only the effect on osteoclastogenesis, but not the tumor cell proliferation or angiogenesis in H1993-induced osteolytic bone lesions. We further showed that loss of TGFBR2 in osteoclasts decreased the expression and secretion of CTHRC1, which stimulated bFGF, possibly through promoting Wnt/β-catenin signaling. Functionally, bFGF stimulated osteoclastogenesis but inhibited osteoblastogenesis. CTHRC1 was able to stimulate H1993 cell proliferation, possibly through non-β-catenin signaling and osteoblastogenesis. Our data fill gaps in our knowledge on how CTHRC1 from osteoclasts regulates bFGF expression in osteoblasts and, thus, contributes to understanding lesion development.

Our in vivo studies revealed the potential commonalities in osteolytic bone lesion development induced by breast, prostate, and NSCLC cancers. For example, bone lesion development was inhibited in all mice with specific loss of *Tgfbr2* in myeloid lineage cells. bFGF was one potential mediator for this effect, and the decreased bone lesion area for all three cancer types correlated with decreases in number of osteoclasts, angiogenesis, and the proliferation of cancer cells. On the other hand, we also found cancer cell type-specific effects. For example, bFGF rescued the proliferation of breast and prostate cancers, as well as angiogenesis and cancer-associated fibroblasts in prostate cancer. However, only the number of osteoclasts was rescued by bFGF in H1993-induced bone lesions, and bFGF did induce osteoclastogenesis in vitro. The proliferation of cancer cells, including H1993 NSCLC cells, was not directly affected by bFGF, suggesting the indirect effect from the tumor microenvironment. However, whether the change of osteoclastogenesis is a cause or a result will need further studies. We point out the caveat that *LysM^Cre^* targets the entire myeloid lineage, which includes not only osteoclasts, but also neutrophils, macrophages, basophils, eosinophils, megakaryocytes, erythrocytes, and mast cells. The mechanisms by which TGF-β signaling in these cells regulates bFGF or other factors could be unique, and were not addressed. The H1993-injected tibiae were not analyzed at 3D level using microCT to further confirm the bone lesions analyzed at 2D using MetaMorph. However, previous studies with both analyses showed reasonable consistency between the cancer-induced bone lesions and the ratios of bone volume to total volume [20,21,37].

Our data demonstrate that CTHRC1 from osteoclasts promoted the Wnt/β-catenin pathway in primary osteoblasts which, in turn, promoted bFGF expression. We found different kinetics of bFGF regulation between CTHRC1 and Wnt3a. Induction of bFGF was about 2 h earlier by Wnt3a CM (conditioned medium) than by CTHRC1. This data further confirmed that Wnt3a directly, but CTHRC1 indirectly, activates β-catenin. In addition, induction of bFGF lasted 48 h by CTHRC1, but only up to 24 h by Wnt3a CM. This is possibly due to the short half-life of Wnt3a [38], or the exhaustion of the nutrient in CM. On the other hand, effects of recombinant CTHRC1 protein had been often observed for up to 6 days [29]. We acknowledge that, due to a lack of well-recognized markers for the Wnt/PCP pathway, LGK794 was chosen for its ability to inhibit Wnt secretion without increasing cytotoxicity. At the transcriptional level, in addition to the decreased CTHRC1, loss of TGFBR2 in osteoclasts caused them to express less SOST. However, validation at the protein level is needed to warrant further investigations of SOST. In addition, most of the literature supported the idea that SOST is mainly expressed in mesenchymal lineage osteoblasts and osteocytes [39,40,41].

Functionally, CTHRC1 and bFGF can be involved in a positive feedback loop, where bFGF from osteoblasts promotes the maturation of osteoclasts, which promotes CTHRC1 expression and secretion which, in turn, promotes osteoblast production of bFGF. On the other hand, we showed for the first time that CTHRC1 and bFGF had antagonizing effects in osteoblastogenesis. The effects on osteoblastogenesis by the combination treatment of CTHRC1 and bFGF were dose-dependent. However, these in vitro data could not be confirmed in H1993-injected mouse tibiae because, in our in vivo osteolytic bone lesions, few osteoblasts could be identified. We also point out that the ALP and TRAP staining, as the readouts for osteoblastogenesis and osteoclastogenesis, respectively, include cells at various differentiation stages. Another caveat is that we were not able to compare the effects of osteoclasts CM from our Flox and KO mice on bFGF expression in osteoblasts. Since the effect of CTHRC1 is dose-dependent, 100 ng/mL is the minimum dose at which we could consistently observe the significant effects in osteoblasts. Large amounts of CM from primary cultured osteoclasts (from mice) need to be generated and concentrated to reach the dose. Furthermore, CTRHC1 could directly stimulate H1993 cell growth. Taken together, the roles of CTHRC1 and bFGF in osteoblasts, osteoclasts, and cancer cells contribute to the complexity of bone lesion development.

Various approaches to blocking TGF-β or its downstream signaling components have shown effectiveness in preclinical animal models of many types of cancer, including NSCLC, but not in clinical trials [15,42,43,44,45]. The pivotal roles of cell autonomous and non-autonomous (from the tumor microenvironment) TGF-β signaling in tumor initiation, progression, metastases, and drug resistance make such signaling a promising candidate for combination therapies against cancers, and an important area for translational research [15,16,46,47,48,49,50,51,52,53,54]. Our approach has been to identify druggable TGF-β signaling downstream mediators, such as cytokines or growth factors that promote bone metastasis. Consistent with our previous studies [20,21], we found that TGF-β signaling in osteoclasts promoted bone lesion development in NSCLC cells such as H1993. We propose that this osteoclastic TGF-β signaling effect occurs regardless of cancer types. These findings will open up new avenues for targeting of bFGF in single or combination therapies against osteolytic cancer bone metastases, including NSCLC. Importantly, our studies highlighted that CTHRC1 promotes the expression of bFGF only in osteoblasts, in a Wnt/β-catenin-dependent manner.

## 4. Materials and Methods

### 4.1. Cells Cultures and Animals

H1993 NSCLC cells were obtained from ATCC and maintained in RPMI1640 (Gibco) supplemented with 10% FBS. These cells were periodically checked for mycoplasma contamination, and mycoplasma-free cells were used for experiments.

*Tgfbr2^floxE2/floxE2^* and *Tgfbr2^LysMCre^* knockout (KO) mice were developed and maintained in Rag^−/−^ immune-deficient mice, which have been characterized and described previously [20]. The research using these mice has been approved by Van Andel Research Institute (VARI) Institutional Animal Care and Use Committee, protocols 13-01-004 and 13-01-005, which were approved on 18 January 2013, and 16-01-004 and 16-01-005, which were approved on 28 January 2016. These protocols have been renewed annually.

Mouse bone marrow cells were extracted from the long bones of 5- to 6-week-old female mice [20,21]. For primary osteoblast cultures, bone marrow cells were cultured in α-MEM with 10% FBS for 3 d and then in α-MEM/10% FBS with addition of 50 μg/mL vitamin C for another 7 d. These cells were then ready for experimental treatment, such as addition of recombinant CTHRC1 protein (100 ng/mL; Sino Biological, Wayne, PA, USA) or LGK-974 (1 nM/mL; Cellagen Technology, San Diego, CA, USA). Alkaline phosphatase (ALP) staining was used to show the differentiation of osteoblasts, and crystal violet staining was used to show cell growth. For primary osteoclast cultures, bone marrow cells were cultured in α-MEM/10% FBS with addition of 30 ng/mL RANKL (R&D) and 25 ng/mL mCSF (Sigma-Aldrich, St. Louis, MO, USA) for 6 d. Tartrate-resistant acid phosphatase (TRAP) staining was used to show the differentiation of osteoclasts, and crystal violet staining was also used to show proliferating cells. Conditioned medium was collected by starving osteoclasts for 24 h in α-MEM, and then concentrated using Ultracel-10k (Sigma-Aldrich).

Wnt3a- and Wnt5a-conditioned media were collected from 5d of incubation of L-Wnt3A and L-Wnt5A cells (acquired from ATCC), respectively, in α-MEM with 5% FBS.

### 4.2. Injections and Radiographic Imaging

One million H1993 cells were suspended in 10 μL of PBS, and injected into the left tibiae of 5- to 6-week-old female mice. The contralateral tibia of each mouse was injected with 10 μL of PBS. Neutralizing antibody for mouse bFGF (bFGF Ab) or IgG as vehicle control (Millipore) were injected into mice as described [20,21]. 

Mice were radiographically imaged weekly (2, 3, and 4 weeks after intratibial injection) using Bioptics piXarray Digital Specimen Radiography (Faxitron Bioptics, Tucson, AZ, USA). The lytic bone lesions each week were scored in a blinded manner by measuring their area using MetaMorph, a quantitative image analysis software program (V7.7.9.0, Molecular Devices, Inc., San Jose, CA, USA).

### 4.3. Histology, Histomorphometry, and Immunohistochemistry

Mouse tibiae were harvested and fixed in 10% neutral-buffered formalin (Sigma-Aldrich) for 4–5 d at 4 °C, followed by decalcification in 14% EDTA for 5–6 d at 4 °C. Paraffin-embedded bone sections (5 μm thick) were stained with hematoxylin and eosin (H&E). Serial sections were stained for TRAP to identify multinucleate osteoclasts [20,21]. Histomorphometric analysis was performed on TRAP-stained sections for tumor burden, and the number of osteoclasts and osteoblasts were counted using Bioquant system imaging software (V18.2.60, Nashville, TN, USA).

Paraffin-embedded sections were stained using antibodies listed in Table S1. As previously described [20,21], CD31 or Ki67 was quantified on IHC-stained mouse tissue sections using ImageJ software (V1.51, Bethesda, MD, USA).

### 4.4. Protein Extraction and Western Blots

Harvested mouse tibiae were snap-frozen in liquid nitrogen, and homogenized using FastPrep-24 (MP Biomedicals, Santa Ana, CA, USA). Cells grown in culture were lysed using RIPA supplemented with proteases and phosphatase inhibitors. Further cell fractionation was used to isolate the cytoplasmic or nucleic proteins, as needed.

For Western blots, 10–20 μg of total protein was quantified and separated by SDS-polyacrylamide gel electrophoresis, then transferred to polyvinylidene difluoride membranes. All membranes were blocked in 5% BSA-TBST, and incubated with primary antibodies overnight at 4 °C, followed by incubation with HRP-conjugated secondary antibodies (Santa Cruz Biotechnology, Santa Cruz, CA, USA). Blots were developed with an enhanced chemiluminescence substrate mixture. The antibodies used are listed in Table S1.

### 4.5. RNA Extraction and Quantitative Real-Time RT-PCR (qRT-PCR)

Total RNA from frozen mouse tibiae or from cells grown in culture was extracted using TRIzol (Invitrogen, Carlsbad, CA, USA). Complementary DNA (cDNA) was synthesized using the SuperScript First-Strand Synthesis System (Invitrogen), and qRT-PCR was performed using SYBR Green Supermix (Bio-Rad, Hercules, CA, USA) on the ABI machine. Primers were designed and synthesized by Integrated DNA Technologies (IDT, Coralville, IA, USA). The sequences of the primers used in this manuscript were listed in Table S2.

### 4.6. Statistical Analysis

Longitudinal bone lesions were analyzed via a linear mixed-effect model. Linear contrasts with a false discovery rate correction were used to test for significant differences in bone lesion growth rates, and bootstrap hypothesis testing with 1000 resampled datasets was used to test for differences in total lesion area between groups at specific time points. For experiments without repeated measures, Student’s *t*-test or ANOVA was used for experiments involving more than two groups. For all analyses, *p* < 0.05, two-tailed was considered significant.

## 5. Conclusions

Loss of myeloid-specific TGF-β signaling decreases CTHRC1 secretion from osteoclasts to downregulate bFGF expression in osteoblasts, thereby inhibiting the H1993-induced osteolytic bone lesion development.

## Supplementary Materials

The following are available online at http://www.mdpi.com/2072-6694/10/12/463/s1, Figure S1. Mouse bFGF expression was decreased in *Tgfbr2^LysMCre^* KO tibiae injected with H1993 cells; Figure S2. bFGF had no effects on proliferation or angiogenesis in H1993 tumors in tibiae; Figure S3. bFGF induced osteoclastogeneis. Representative images of osteoclast differentiation from mouse bone marrow; Figure S4. Relative expressions of clastokines of osteoclasts from *Tgfbr2^floxE2/floxE2^* and *Tgfbr2^LysMCre^* KO mice; Figure S5. Effects of CTHRC1 on H1993 cells. A. CTHRC1 did not increase bFGF expression. Table S1. Information of the antibodies used; Table S2. List of the primers’ sequences.
