# Supplementary material for: Loss of Myeloid-Specific TGF-β Signaling Decreases CTHRC1 to Downregulate bFGF and the Development of H1993-Induced Osteolytic Bone Lesions
Source: Cancers (Basel). 2018 Nov 22;10(12):463. doi: 10.3390/cancers10120463 (PMC6315699; doi:10.3390/cancers10120463)
Supplement: Supplementary file 1 [file cancers-10-00463-s001.pdf]

Supplemental Materials: Myeloid-Specific TGF-β Signaling Stimulates CTHRC1 and Non-Small Cell Lung Cancer Bone Lesions in a bFGF-Dependent Manner

Sourik S. Ganguly, Paul G. Daft, Jingchen Cao, Xiangqi Meng, Zhendong A. Zhong, Alexandra Vander Ark, Austin Meadows, Zach Madaj, Bart Williams and Xiaohong Li

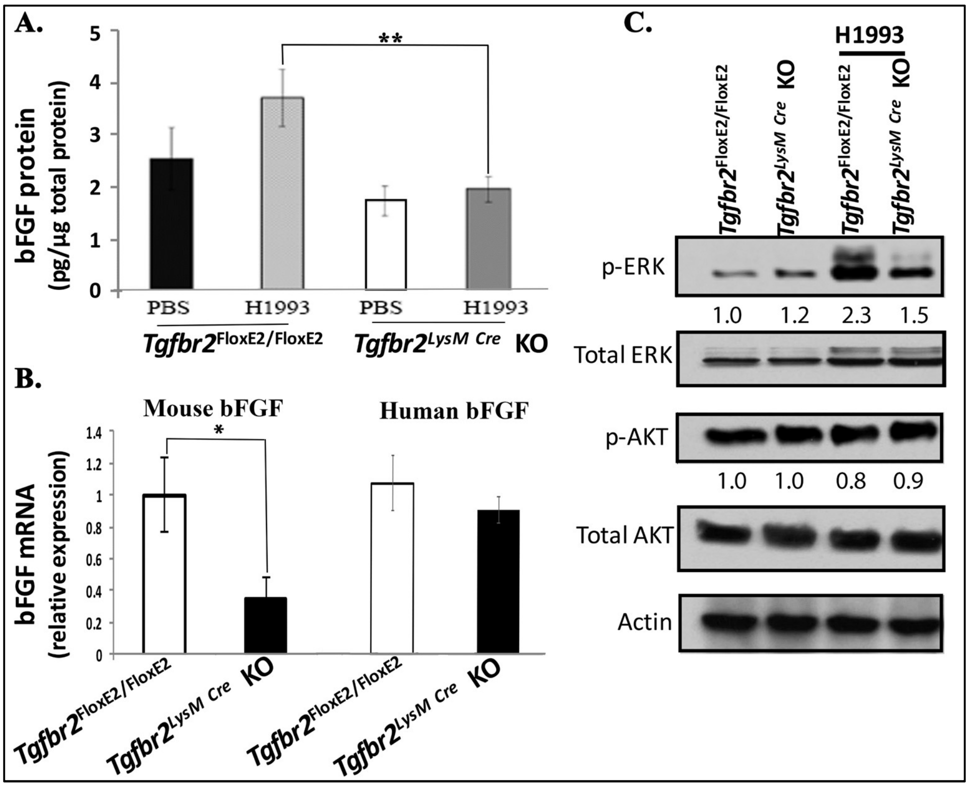

Figure S1. Mouse bFGF expression was decreased in *Tgfbr2*^{LysMCre} KO tibiae injected with H1993 cells. **A.** ELISA of bFGF from H1993-injected mouse tibiae. **B.** qRT-PCR using mouse species-specific bFGF primers. **C.** Representative western blot using H1993-injected and non-injected mouse tibiae. Numbers under each blot are relative quantification of the densities normalized to actin. In the relative quantification for p-ERK and p-AKT, the densities were further normalized to the respective total proteins. Mean ± S.D., $n \geq 3$ per group. *$p \leq 0.05$, **$p \leq 0.01$by Student's *t*-test.

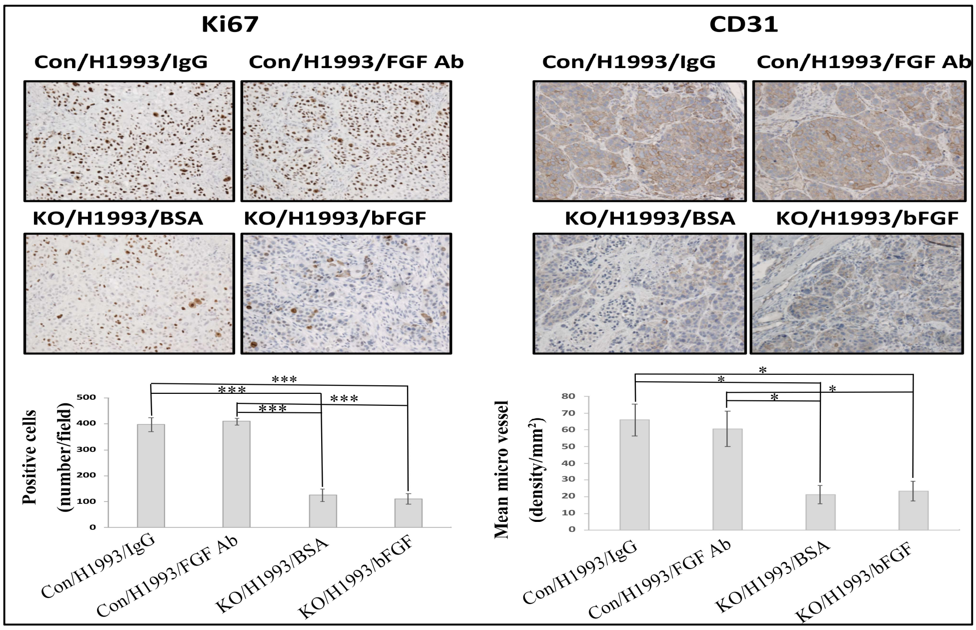

Figure S2. bFGF had no effects on proliferation or angiogenesis in H1993 tumors in tibiae. Representative images of IHC staining for Ki67 and CD31 (20×) and quantifications of the positive staining. Mean ± S.D., $n \geq 7$. ***$p < 0.001$, *$p \leq 0.05$, by ANOVA.

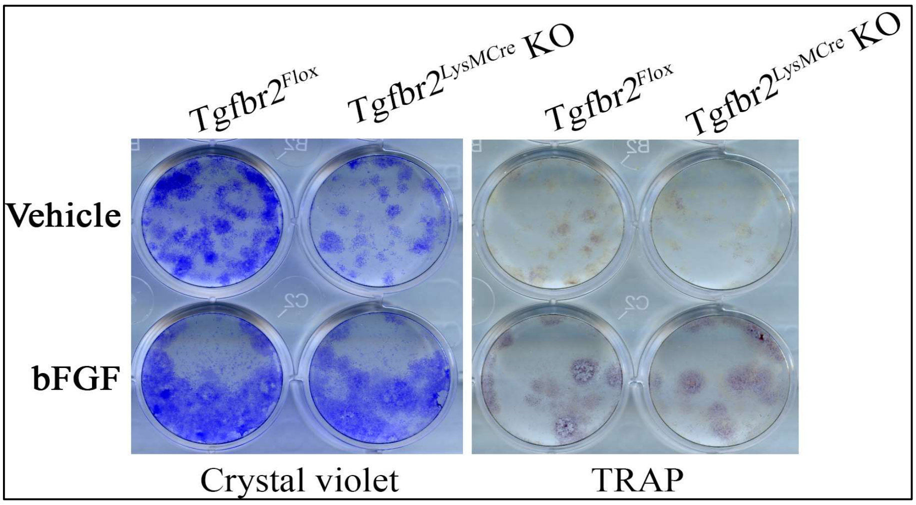

Figure S3. bFGF induced osteoclastogeneis. Representative images of osteoclast differentiation from mouse bone marrow. TRAP staining shows the differentiated osteoclasts, and crystal violet staining shows all cells. Experiments were repeated at least three times with the same results. More than six mice per group were used for collecting bone marrow.

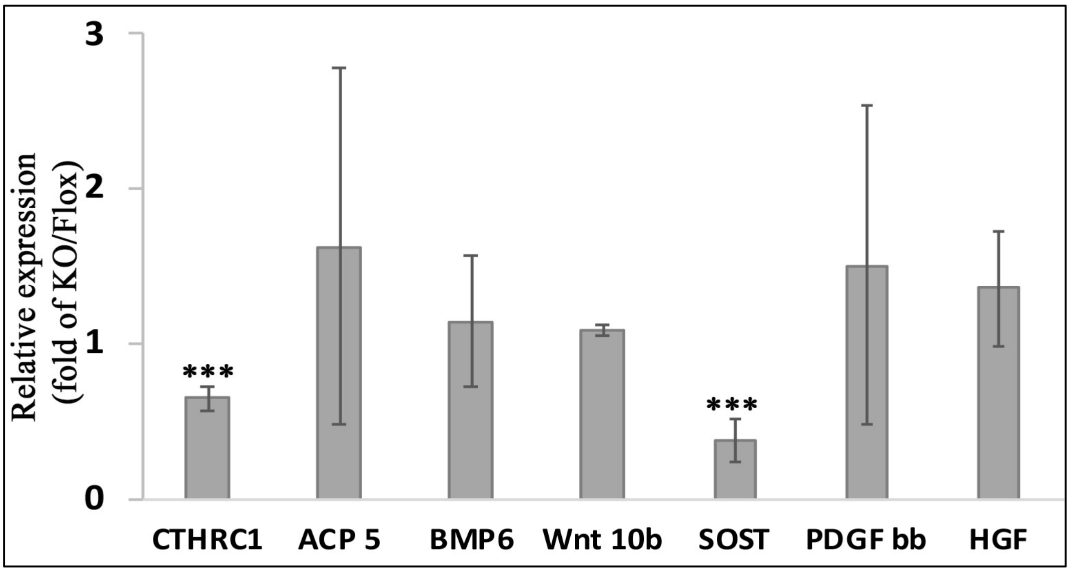

Figure S4. Relative expressions of clastokines of osteoclasts from *Tgfbr2*$^{floxE2/floxE2}$ and *Tgfbr2*^{LysMCre} KO mice. Primary osteoclasts were cultured from *Tgfbr2*^{LysMCre} KOs and littermate controls. *ACP5* is the gene name for TRAP. $n = 3$; three pairs of littermates were used. ***$p < 0.001$, by Student's *t*-test.

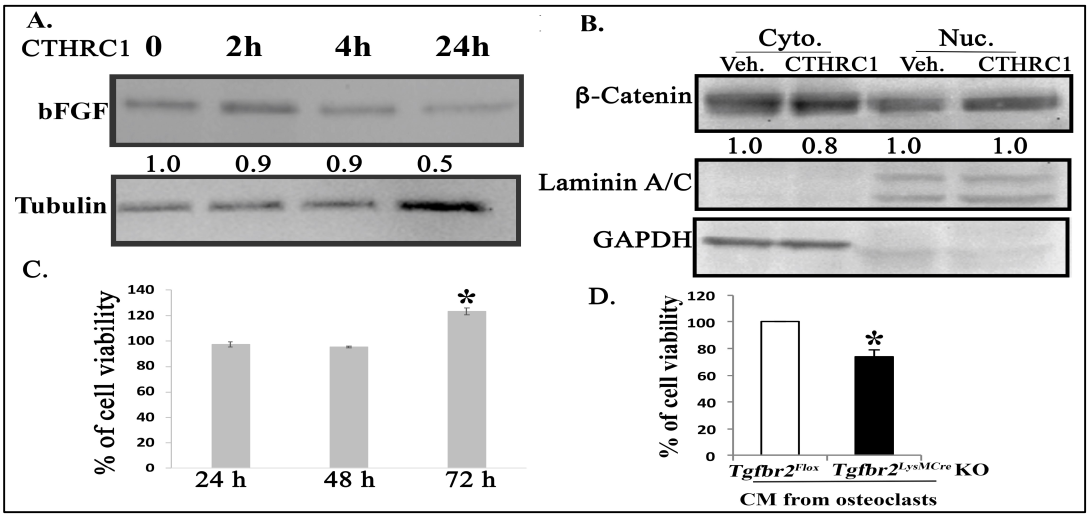

Figure S5. Effects of CTHRC1 on H1993 cells. **A**. CTHRC1 did not increase bFGF expression. H1993 cells were treated with recombinant Cthrc1 (100 ng/mL) for various times and total cell lysates were western blotted for bFGF and tubulin. **B.** CTHRC1 did not change the amount of β-catenin. H1993 cells were treated with recombinant Cthrc1 for 48 h, and cytoplasmic (Cyto.) and nuclear (Nuc.) proteins were extracted for western blotting. Numbers under each blot are relative quantification of the densities normalized to tubulin for total protein, GAPDH for Cyto. proteins, or laminin A/C for Nuc. proteins. **C.** CTHRC1 increased cell viability. H1993 cells were treated with recombinant Cthrc1 for various times. Cell viability is relative to vehicle control at the same time points. **D.** Osteoclast conditioned media from *Tgfbr2*^{LysMCre} KO mice decreased cell viability. H1993 cells were incubated for 24 h with conditioned medium from primary osteoclasts cultured from *Tgfbr2*^{LysMCre} KO and littermate control mice. Relative cell viabilities were analyzed by MTT assay. Mean ± S.D., $n = 3$. *$p \leq 0.05$, by Student's *t*-test. More than 12 mice were used for collecting bone marrow.

Table S1. Information of the antibodies used.

Name	Cat #	Company	Dilution	Application
β-Catenin	9562	Cell Signaling Technology	1:1000	WB
Total AKT	4691S		1:1000	WB
Total ERK	4695S		1:4000	WB
FGFR1	9740		1:1000	WB
p-AKT	4060S		1:1000	WB
p-ERK	4370S		1:1000	WB
DVL2	3216		1:1000	WB
bFGF	05-118	Millipore	1:1000	WB
β-actin	SC-1615	Santa Cruz	1:2000	WB
TGFBR2	SC400		1:500	WB
GAPDH	SC365062		1:10,000	
Tubulin	T9026	Sigma	1:10,000	WB
Ki-67	RM-9106	Thermo Scientific	1:100	IHC
CD31	Ab28364	Abcam	1:1000	IHC
CTHRC1	Ab192778		1:200	WB

IHC: Immunohistochemistry; WB: Western Blot.

Table S2. List of the primers' sequences.

Gene	Species	Sequence (5′-3′)
bFGF	Mouse	Forward: GACCCACACGTCAAACTACA
		Reverse: GCCGTCCATCTTCCTTCATAG
	Human	Forward: GACCCTCACATCAAGCTACAA
		Reverse: AGCCAGTAATCTTCCATCTTCC
CTHRC1	Mouse	Forward: CTGCTACAGTTGTCCGCACC
		Reverse: GGTCCTTGTAGACACATTCCATT
GAPDH	Mouse	Forward: GTACACTCCAGGTCTACCACC
		Reverse: TCCTCAGTGTAGCCCAAGA
	Human	Forward: GTGGTCTCCTCTGACTTCAAC
		Reverse: CCTGTTGCTGTAGCCAAATTC
ACP5	Mouse	Forward: GCAGTATCTTCAGGACGAGAAC
		Reverse: TCCATAGTGAAACCGCAAGTAG
BMP6	Mouse	Forward: CGCACCAACCAAACTGAATG
		Reverse: CACAAGCTCTCACGACCATATT
WNT10b	Mouse	Forward: GCTGAGCAGAGCCATCTTTAT
		Reverse: CTCAAAGTAAACCAGCTCTCCA
SOST	Mouse	Forward: GGCAAGCCTTCAGGAATGA
		Reverse: GGTTCATGGTCTGGTTGTTCT
PDGF	Mouse	Forward: TGCACAGAGACTCCGTAGAT
		Reverse: CTCGAGATGAGCTTTCCAACTC
HGF	Mouse	Forward: CTGGCCTCTTCTATGGCTATTAC
		Reverse: ATCCCAAATCGT CCTGGTATTT
